# Mixtures of Algal Oil and Terrestrial Oils in Diets of Tiger Puffer (*Takifugu rubripes*)

**DOI:** 10.3390/ani15091187

**Published:** 2025-04-22

**Authors:** Lu Zhang, Haoxuan Li, Ziling Song, Qingyan Gao, Chenchen Bian, Qiang Ma, Yuliang Wei, Mengqing Liang, Houguo Xu

**Affiliations:** 1 College of Fisheries and Life Sciences, Shanghai Ocean University, 999 Huchenghuan Road, Shanghai 201306, China; 2 State Key Laboratory of Mariculture Biobreeding and Sustainable Goods, Yellow Sea Fisheries Research Institute, Chinese Academy of Fishery Sciences, 106 Nanjing Road, Qingdao 266071, China

**Keywords:** marine fish, lipid source, feed ingredient, LC-PUFA sparing, sustainable aquaculture

## Abstract

The n-3 long-chain polyunsaturated fatty acids (n-3 LC-PUFAs) have a key role in maintaining fish growth and health. However, fish oil (FO), the main source of n-3 LC-PUFAs, is in relative shortage due to the rapid development of the aquaculture industry. In this study, we investigated the efficacy of replacing fish oil with mixtures of algal oil (AO) from *Schizochytrium* sp. and terrestrially sourced oils (animal oil poultry oil (PO) or vegetable oil rapeseed oil (RO)) in the diets of juvenile tiger puffer (average initial body weight 23.8 ± 1.51 g). An 8-week feeding trial was conducted using three experimental diets: a control diet containing 6% added FO (control FO-C) and two diets with 3% AO + 3% PO or RO (groups AO+PO and AO+RO, respectively), replacing FO. Each diet was fed to triplicate tanks with 25 fish in each tank. The weight gain, feed conversion ratio, body composition, and serum biochemical parameters were not significantly different among the three groups, except that the AO+PO group had a significantly lower muscle lipid content than the other two groups. The AO-added diets significantly increased the DHA content in whole fish, muscle, and liver samples but significantly reduced the EPA content. The oil mixture treatments significantly increased the contents of monounsaturated fatty acid (MUFA) but significantly decreased the contents of saturated fatty acids (SFAs) in the liver and whole fish samples. However, the MUFA and SFA contents in the muscle samples were not significantly different among the dietary groups. The diets with oil mixtures did not affect the hepatic histology but tended to result in the atrophy of intestinal villi. The treatment diets downregulated the hepatic gene expression of proinflammatory cytokines (*il-1β* and *tnf-α*) and the fibrosis marker gene, *acta2*. However, the AO+PO diet inhibited the intestinal gene expression of the tight junction protein, *claudin 18*. In the muscle, the treatment diets upregulated the expression of genes related to cell differentiation and apoptosis (*myod*, *myog*, *myf6*, *myf5*, *bcl-2*, and *bax*). In conclusion, *Schizochytrium* sp. oil in combination with terrestrial oils (poultry oil or rapeseed oil) can be an effective alternative to fish oil in the diets of tiger puffer, but the mixing strategy may be better modified in consideration of intestinal health.

## Simple Summary

With the rapid development of the aquaculture industry, finding alternative lipid sources to fish oil has become a key task for the aquafeed sector. Algal oil (AO) derived from *Schizochytrium* sp. is considered a highly promising alternative lipid source due to its rich content of long-chain polyunsaturated fatty acids (LC-PUFAs), particularly docosahexaenoic acid (DHA). Numerous studies have shown that AO can effectively support fish growth performance, immune function, and health status when partially or completely replacing fish oil, but the sole use of AO makes the diets lack saturated fatty acids (SFAs) and monounsaturated fatty acids (MUFAs), which are good energy substrates. This study aims to further investigate the efficacy of AO in diets of tiger puffer (*Takifugu rubripes*), when used in combination with terrestrially sourced oils rich in SFAs and MUFAs.

## 1. Introduction

The n-3 long-chain polyunsaturated fatty acids (n-3 LC-PUFAs), such as docosahexaenoic acid (DHA,22:6n-3), eicosapentaenoic acid (EPA, 20:5n-3), and arachidonic acid (ARA, 20:4n-6), play key roles in regulating fish growth and health [1,2,3]. At present, marine fish oil (FO) is still the main source of DHA and EPA for human consumption and animal feeds. However, the global wild fishery resources have been in continuous decline for nearly three decades, with an average annual decline rate of 1.2% in catch. At the same time, the rapid development of the aquaculture industry has further exacerbated the conflict between FO supply and demand. The development of alternative lipid sources has become an urgent need to support the sustainable development of aquaculture [4].

Terrestrial animal oils, such as poultry oil, beef tallow, and lard, as well as vegetable oils, such as soybean oil, rapeseed oil, and palm oil, have been widely used as partial or complete substitutes for FO due to the advantages of a relatively lower price and stabler supply [5,6,7]. However, these oil sources have deficient n-3 LC-PUFAs and an unbalanced fatty acid composition, which may lead to metabolic disorders in farmed fish and a decrease in the nutritional value of fish fillets in terms of their fatty acid profile [8]. Notably, fish have a limited ability to synthesize DHA and EPA on their own, and the accumulation of n-3 LC-PUFAs in the body of wild fish originally derives from microalgae [9,10,11]. This suggests that microalgae may be an ideal candidate for FO replacement.

In recent years, significant progress has been achieved in the large-scale and economical cultivation of oil-producing microalgae, such as *Schizochytrium* sp. and *Crypthecodinium cohnii*, which contain up to 30–50% of the dry weight as DHA [12,13]. *Schizochytrium* sp. oil, the most commercialized algae oil (AO), has been used as a FO substitute in feeds for some economically important fish species [14,15]. However, the sole use of AO as a lipid source in fish feed may lead to a high cost and a lack of saturated fatty acids (SFAs) and monounsaturated fatty acids (MUFAs), which can be preferentially mobilized as energy substrates. Furthermore, the adequate supply of SFAs and MUFAs in the diet can reduce the catabolic consumption of LC-PUFAs, and thereby spare the LC-PUFAs [16]. Therefore, the combined use of AO and oils rich in SFAs and MUFAs would be a reasonable method to provide lipids for fish feeds. Animal oil, namely poultry oil (PO), and vegetable oil, namely rapeseed oil (RO), are representative terrestrially sourced oils rich in SFAs and MUFAs. The successful replacement of FO by PO and RO, partially or completely, have been demonstrated in several fish species, such as large yellow croaker (*Larimichthys crocea*) (replacing 50% FO) [17], brown trout (*Salmo trutta*) (replacing 100% FO) [18], and largemouth bass (*Micropterus salmoides*) (replacing 100% FO) [19]. Nevertheless, in yellowtail kingfish (*Seriola lalandi*) [20], Atlantic salmon (*Salmo salar*) [21], and rainbow trout (*Oncorhynchus mykiss*) [22], the complete replacement of FO by PO or RO still resulted in decreased growth, impaired health, and more importantly, lowered muscle LC-PUFA contents. In contrast, the combination of AO and PO or RO can meet the energy and cell structure-oriented fatty acid requirements simultaneously and allows more LC-PUFAs to be retained for critical physiological processes, such as cell membrane construction and eicosanoid synthesis [23,24].

Tiger puffer is an economically important species for the fish farming in Asia and is also becoming an important model fish in nutritional research due to the published genome information [25,26,27]. This species has a relatively unique lipid storage pattern, namely a very low lipid content in the muscle and no adipose tissue at all. Alternatively, they store lipids predominantly in the liver [28]. The aim of this study was to comprehensively evaluate the effectiveness of a combination of AO with PO or RO as an alternative to FO in the diets of tiger puffer (*Takifugu rubripes*), in terms of growth performance, health, and fatty acid composition. Tiger puffer is a lean species, with a muscle lipid content of less than 1%. The low lipid content in the muscle may make it easier to deposit adequate LC-PUFAs in the muscle [29]. The results of this study may provide an efficient way to replace the FO in fish diets without compromising the fish growth and muscle LC-PUFA contents.

## 2. Materials and Methods

### 2.1. Experimental Diets

Based on the protein (41%) [30] and lipid (11.5%) (11.5%) [31] requirements of the tiger puffer, three isonitrogenous (appr. 49% crude protein), isolipidic (appr. 11% crude lipid), and isoenergetic (approximately 20.9 kJ/g energy) diets were formulated (Table 1 and Table 2). The experimental diets differed only in their lipid sources, namely, fish oil (FO), algal oil (AO, *Schizochytrium* sp.), poultry oil (PO), and rapeseed oil (RO). Fish oil, *Schizochytrium* sp. oil, poultry oil, and rapeseed oil were purchased from Qingdao Surgreen Bioengineering Co., Ltd. (Qingdao, China), Xiamen Huison Biotech Co., Ltd. (Xiamen, China), Shandong Haiding Agriculture & Animal Husbandry Co., Ltd. (Jinan, China), and Shandong Luhua Group Co., Ltd. (Yantai, China), respectively. The control diet contained 6% added marine FO (FO-C), and the treatment diet contained 3% AO + 3% PO (AO+PO) or 3% AO + 3% RO (AO+RO), replacing 100% of the added FO (accounting for around half of the total lipids). Pellets with 1.0 mm diameters were prepared. The pellets were dried in an oven at 55 °C for 12 h. Finally, all the diets were packaged and stored at −20 °C before being used.

### 2.2. Feeding Procedure and Sampling

In this experiment, juvenile tiger puffer (average initial weight 23.8 ± 1.51 g) was used. The fish were firstly fed a commercial feed for 2 weeks to acclimatize to the experimental environment at the Langya Experiment Base of the Yellow Sea Fisheries Research Institute (Qingdao, Shandong, China). When the feeding trial was started, after fasting for 24 h, the juvenile fish were randomly assigned to nine tanks (polyethylene, 300 L, triplicate tanks for each group, with 25 fish in each tank). The experimental period lasted 8 weeks with daily feeding to apparent satiation (the feeding rate was approximately 2.15% of the fish body weight) twice, at 8:00 and 18:00. The residual feeds and feces were removed daily using a siphoning system. In the tanks, 2/3 of the water was changed each day. The water temperature during the experiment was 27–31 °C; salinity, 28–30; dissolved oxygen, >8 mg/L; pH, 7.6–7.8; light:dark time, 12 h:12 h.

At the end of the feeding process, the fish were first fasted for 24 h before sampling. The weight and survival of the fish in each tank were measured. After anesthesia with eugenol (eugenol:water = 1:10,000, *v*/*v*), three fish were randomly collected from each tank for the analysis of their proximate composition. In addition, eight fish were randomly selected from each tank for tissue sampling. A 1 mL syringe was used to collect the blood samples from the anal fin site. The blood was put into a 1.5 mL centrifuge tube, and after 4 h (4 °C), it was then centrifuged (10 min, 4000× *g*, 4 °C) in order to isolate the supernatant as a serum sample. From each fish, two pieces of dorsal muscle, two pieces of apical liver tissue, and one piece of midgut (approximately 1.0 cm) were collected for the real-time quantitative polymerase chain reaction (RT-qPCR). The samples for the RT-qPCR were frozen immediately with liquid nitrogen and then transferred to storage at −76 °C prior to use. The tissue samples for the analysis of the proximate composition were stored at −20 °C prior to use. All the sampling protocols in this study, as well as all the fish rearing practices, were reviewed and approved by the Animal Care and Use Committee of Yellow Sea Fisheries Research Institute.

### 2.3. Proximate Composition Analysis for Fish and Diets

The analyses of the proximate compositions of the experimental diets, whole fish bodies, muscles, and livers were performed using the methods of the Association of Official Analytical Chemists (AOAC, 1995) [32]. In general, the analysis of the moisture content was conducted by drying the diets and fish samples to a constant weight at 105 °C; the protein content was analyzed by measuring the nitrogen content (N × 6.25) according to the Kjeldahl method; the lipid content in the diets and the whole fish bodies was analyzed with petroleum ether extraction according to the Soxhlet method (but the lipid in the muscle and liver samples was extracted according to the chloroform–methanol method); and the ash content was measured with the incineration method using a muffle furnace (550 °C, 8 h).

### 2.4. Biochemical Parameters of Serum

Serum samples from three fish in each tank were combined. Using commercial kits from the Nanjing Jiancheng Bioengineering Institute (Nanjing, China), the concentrations of the total cholesterol (TC), total triglyceride (TG), total bile acid (TBA), malondialdehyde (MDA), low-density lipoprotein cholesterol (LDL-C), and high-density lipoprotein cholesterol (HDL-C) in the serum were determined.

### 2.5. Fatty Acid Composition Histological Structure

The fatty acid profiles of the oils, diets, muscle samples, and liver samples were analyzed using the gas chromatography method, which was equipped with a quartz capillary column (SH-RT−2560, 100 m × 0.25 mm × 0.20 μm) and a flame ionization detector (GC2010 pro, Shimadzu, Kyoto, Japan). The total lipids were first extracted from the oil, diet, and tissue samples according to the chloroform–methanol method. Subsequently, the fatty acids were then saponified and methylated with boron trifluoride and KOH–methanol solutions. The fatty acid concentrations were expressed as % total fatty acids (TFAs). More details about the method are available in previous publications [33].

### 2.6. Histological Structure

Tissue (intestine and liver) samples from one fish from each tank were used for the histological analyses, which were performed following the methods mentioned in our previous papers [34]. The slices made were investigated and photographed with a Digital Slide Scanner (3DHISTECH Ltd., Budapest, Hungary). All the slices were analyzed using ImageJ 1.53c (Wayne Rasband National Institutes of Health, Bethesda, MD, USA) for the statistical analysis.

### 2.7. cDNA Preparation and Real-Time Quantitative Polymerase Chain Reaction (RT-qPCR) Analysis

The total RNA was extracted from the liver, muscle, and midgut samples (six individuals per tank) using RNAiso Plus (TaKaRa Biotechnology (Dalian) Co., Ltd., Dalian, China). The RNA purity was assessed using a Colibri microvolume spectrophotometer (Titertek Berthold, Pforzheim, Germany), based on the A260/A280 ratio (1.8–2.0). Reverse transcription was conducted with the PrimeScript™ RT Reagent Kit with a gDNA Eraser (TaKaRa, Dalian, China), as per the manufacturer’s instructions. Gene-specific primers for the target and reference genes (*rpl13* and *rpl19*) [35] were synthesized by TsingKe Biological Technology Co., Ltd. (Qingdao, China) (Table 3). The amplification efficiency of all the primers, which was determined by standard curves based on a 6-step, 8-fold dilution series of the target template, varied between 95% and 110%, with linear regression coefficients of (R^2^) > 0.99. The RT-qPCR was conducted using SYBR^®^ Premix Ex Taq TM (TaKaRa Biotechnology (Dalian) Co., Ltd., Dalian, China) and a quantitative thermal cycler (Roche LightCycler 96, Basel, Switzerland). The reaction mixture consisted of 2 μL of the cDNA template, 10 μL of SYBR^®^ Premix Ex Taq TM (2×), 0.8 μL of the forward primer (10 μM), 0.8 μL of the reverse primer (10 μM), and 6.4 μL of sterilized water. The thermal cycling program was as follows: 95 °C for 30 s, followed by 40 cycles of “95 °C for 5 s, 57 °C for 30 s, and 72 °C for 30 s”. After the amplification phase, a melting curve analysis was performed (from 65 °C to 97 °C, incrementing by 6.4 °C per min) to confirm the specificity of the products. Each sample was run in triplicate. Gene expression levels were calculated using the RT-qPCR method: 2^−ΔΔCt^ [36].

### 2.8. Calculation and Statistical Analysis

Weight gain (g) = final body weight-initial body weight. Weight gain ratio (%) = (final body weight-initial body weight)/initial body weight × 100. Specific growth rate = (ln(final weight)-ln(initial weight))/days of experiment × 100. Feed conversion ratio = (final weight-initial weight)/total feed intake. Survival = final fish number/initial fish number × 100. Hepatosomatic index = liver weight/body weight × 100. Viscerosomatic index = viscera weight/body weight × 100. Condition factor = body weight/body length^3^ × 100.

All the data were analyzed by a one-way analysis of variance (ANOVA) in SPSS 27.0.1 (Armonk, NY, USA) for Windows. All the percentage data were arcsine-transformed before the analysis. A Levene test was used to test the homogeneity of the variance. Significant differences between the group means were tested by Tukey’s multiple range test. The significance level was *p* < 0.05. All the results were presented as the means of tanks ± standard errors (SEs).

## 3. Results

### 3.1. Growth Performance, Somatic Indices, and Body Composition

No significant differences (*p* > 0.05) were observed among the groups in terms of the final body weight, weight gain, specific growth rate, feed conversion ratio, and survival (Table 4). No significant differences (*p* > 0.05) were found among the groups in terms of the hepatosomatic index, viscerosomatic index, and condition factor.

The body composition parameters were not significantly different among the three groups, except that the AO+PO group had a significantly (*p* < 0.05) lower muscle lipid content than the other two groups (Table 5).

### 3.2. Serum Biochemical Parameters

The LDL-C levels in the AO+PO group were significantly (*p* < 0.05) higher than those in the FO-C and AO+RO groups (Table 6). No significant difference was observed in other serum biochemical parameters among the three groups.

### 3.3. Fatty Acid Profiles in the Whole Fish, Muscle, and Liver Samples

In the whole fish samples, compared to the FO-C group, the AO+PO and AO+RO groups had significantly increased levels of n-3 PUFA, n-6 PUFA, and MUFA, while the SFA content was significantly (*p* < 0.05) reduced (Table 7). Specifically, compared to the FO-C group, the diets with oil mixtures significantly (*p* < 0.05) increased the contents of DHA, 18:2n-6, and 18:1n-9 but significantly (*p* < 0.05) decreased the contents of EPA, 22:1n-9, 20:1n-9, 16:1n-7, 20:0, 16:0, and 14:0. The 18:3n-3 content in the AO+RO group was significantly higher than that in the other two groups.

In the liver samples, compared to the FO-C group, the AO+PO and AO+RO groups had significantly (*p* < 0.05) increased levels of n-3 PUFA, n-6 PUFA, and MUFA, while the SFA content was significantly (*p* < 0.05) reduced (Table 8). Specifically, compared to the FO-C group, the diets with oil mixtures significantly (*p* < 0.05) increased the contents of DHA and 18:2n-6 but significantly (*p* < 0.05) decreased the contents of EPA, 22:5n-3, 22:1n-9, 20:1n-9, 16:1n-7, 20:0, 16:0, and 14:0. Additionally, the contents of 18:3n-3 and 18:1n-9 in the AO+PO group were significantly (*p* < 0.05) higher than that in the other two groups.

In the muscle samples, compared to the FO-C group, the AO+PO and AO+RO groups showed no significant (*p* > 0.05) effects on the contents of n-3 PUFA, n-6 PUFA, MUFA and SFA (Table 9). The DHA content was significantly (*p* < 0.05) higher in the AO+RO group compared to the other two groups, while the 20:0 and 18:3n-3 contents were significantly (*p* < 0.05) lower in the AO+PO group compared to the other two groups. In addition, compared to the FO-C group, the diets with oil mixtures significantly (*p* < 0.05) decreased the contents of EPA, 22:5n-3, ARA, 16:1n-7, and 14:0.

### 3.4. Histological Structure of Tissues

**Figure 1:**
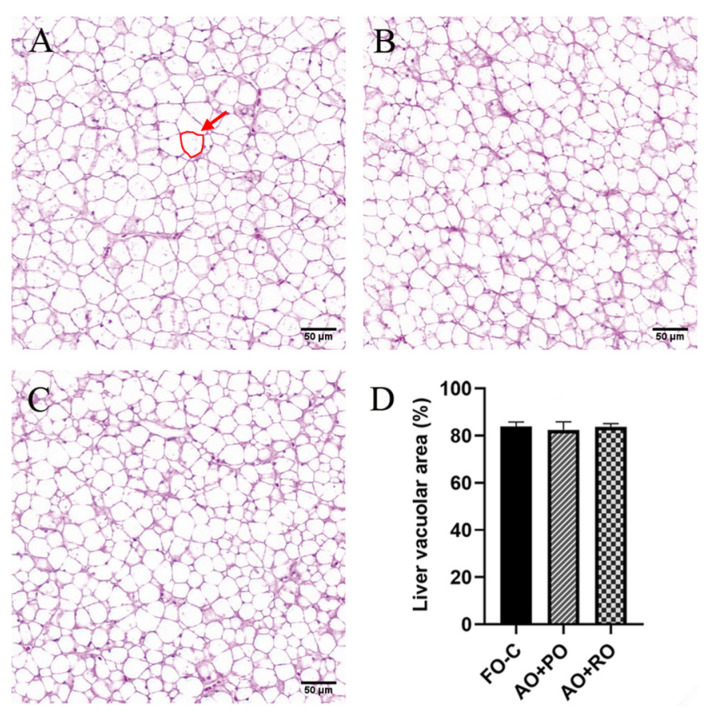
Effects of different dietary lipid sources on liver histology of tiger puffer (*Takifugu rubripes*). (**A**–**C**) show the H&E staining of liver transversal slices (20×) of the FO-C, AO+PO, and AO+SO groups of tiger puffer, respectively; (**D**) shows the liver vacuolar area in tiger puffer, with six view fields (20×) analyzed per slice; The area marked by the red circle represents the vacuolar area of a single vacuole.

**Figure 2:**
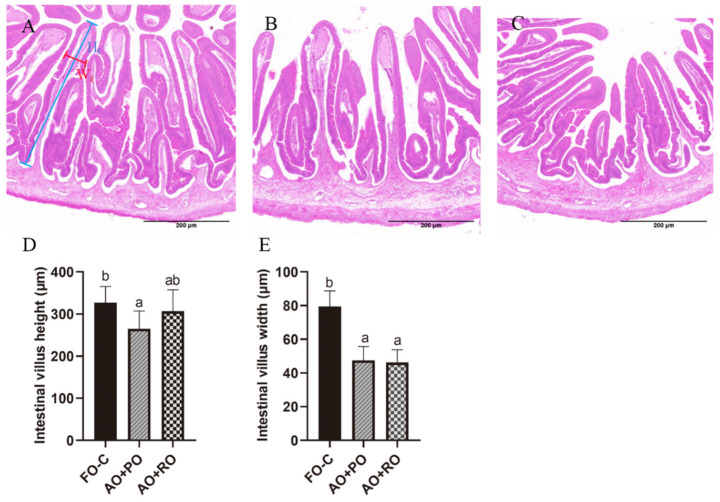
Effects of different dietary lipid sources on the intestinal histology of tiger puffer (*Takifugu rubripes*). (**A**–**C**) show the H&E staining of intestinal transversal slices (5×) of the FO-C, AO+PO, and AO+SO groups, respectively. (**D**,**E**) show the intestinal villus height and width, respectively, with six randomly selected intestine villus analyzed per slice; The blue and red lines show the height (H_I_) and width (W_I_) of intestine villus, respectively. Data bars not sharing a same letter are significantly (*p* < 0.05) different.

The experimental diets had no significant effects on the hepatic vacuolar area (*p* > 0.05) (Figure 1). However, significant damage to intestinal structural integrity by the AO+PO and AO+RO diets was observed, characterized by reduced villus height and width (*p* < 0.05) (Figure 2).

### 3.5. Gene Expression

#### 3.5.1. Liver Fibrosis and Inflammation Related Gene Expression

**Figure 3:**
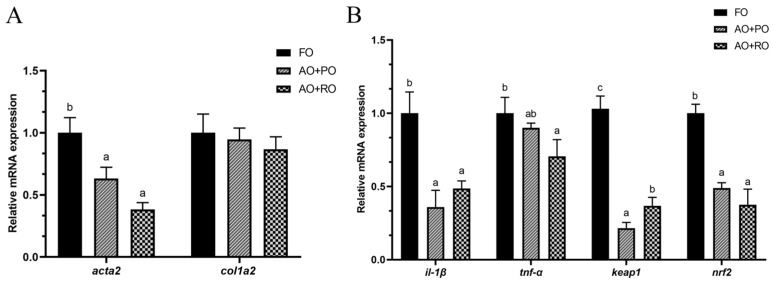
Effects of different dietary lipid sources on the mRNA expression levels of liver fibrosis (**A**) and inflammation (**B**) related gene expression in the liver of tiger puffer (*Takifugu rubripes*). Data are presented as mean ± SEM (n = 3). According to Tukey’s test, bars not sharing a same letter are significantly (*p* < 0.05) different.

Compared to the FO-C group, the AO+PO and AO+RO groups significantly (*p* < 0.05) reduced the hepatic expression of *acta2*, but showed no significant (*p* > 0.05) effect on *col1a2* expression (Figure 3A). Compared to the FO-C group, the AO+PO and AO+RO groups significantly (*p* < 0.05) downregulated the hepatic expression of *il-1β* and *nrf2*. The expression levels of *tnf-α* and *keap1* in the FO-C group was significantly (*p* < 0.05) higher than that in the treatment groups (Figure 3B).

#### 3.5.2. Intestinal Inflammation and Intestinal Barrier-Related Gene Expression

**Figure 4:**
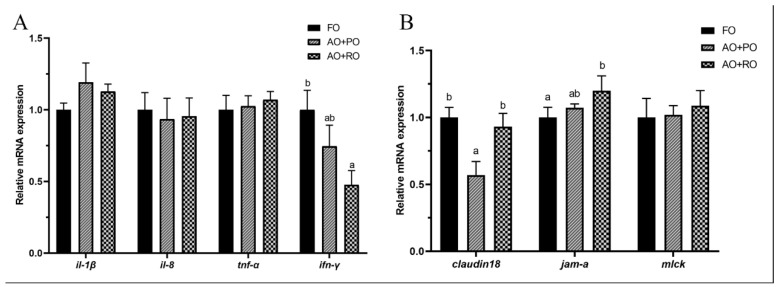
Effects of different dietary lipid sources on the mRNA expression levels of intestinal inflammation (**A**) and intestinal barrier-related (**B**) gene expression in the intestine of tiger puffer (*Takifugu rubripes*). Data are presented as mean ± SEM (n = 3). According to Tukey’s test, bars not sharing a same letter are significantly (*p* < 0.05) different.

Compared to the FO-C group, the AO+PO and AO+RO groups had no significant (*p* > 0.05) effects on the expression levels of *il-1β*, *il-8*, and *tnf-α*, but diet AO+RO significantly (*p* < 0.05) downregulated the expression of *ifn-γ* (Figure 4A). Additionally, the expression levels of *jam-a* were upregulated by diet AO+RO, while the expression levels of *claudin18* in the AO+PO group was significantly (*p* < 0.05) lower than that in the other two groups. Meanwhile, the expression level of *mlck* showed no significant (*p* > 0.05) differences among the treatment groups (Figure 4B).

#### 3.5.3. Muscle Differentiation Apoptosis Related Gene Expression

**Figure 5:**
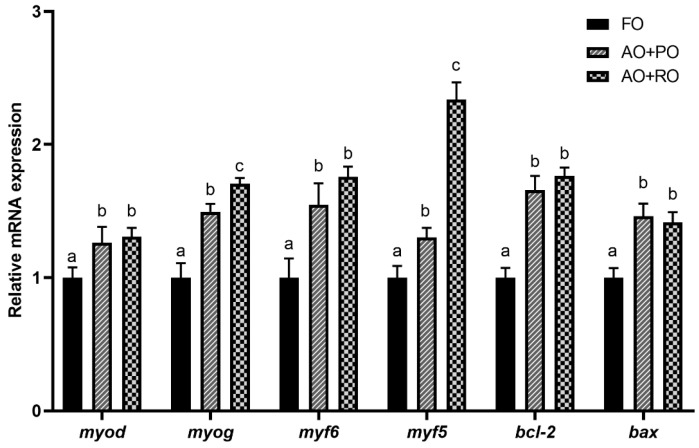
Effects of different dietary lipid sources on the mRNA expression levels of cell differentiation- and apoptosis-related genes in the muscles of tiger puffer (*Takifugu rubripes*). Data are presented as mean ± SEM (n = 3). According to Tukey’s test, bars not sharing the same letter are significantly (*p* < 0.05) different.

Compared to the FO-C group, the diets with oil mixtures exhibited significantly (*p* < 0.05) upregulated expression levels of *myod*, *myf6*, *bcl-2*, and *bax*. The expression levels of *myog* and *myf5* in the AO+RO group were significantly (*p* < 0.05) higher than those in the other two groups (Figure 5).

## 4. Discussion

Fish oil (FO) replacement by alternative lipid sources has been an urgent task for the aquafeed industry. However, whether FO can be successfully replaced for a certain fish species is determined by many factors. One of them is the capacity of the fish to synthesize LC-PUFAs. It is widely recognized that freshwater and euryhaline fish species can synthesize LC-PUFAs from C18 precursors, such as 18:3n-3 and 18:2n-6, whereas most marine carnivorous fish lack this ability. This disparity is attributed to the abundance of LC-PUFAs in marine ecosystems, which are primarily derived from lower trophic organisms, such as microbes, algae, and invertebrates. As a result, LC-PUFA bioconversion pathways in marine species have largely become redundant [37,38,39,40]. In contrast, freshwater and anadromous species, which face a relative scarcity of dietary LC-PUFAs, have evolved a greater capacity for LC-PUFA biosynthesis. This hypothesis makes algae one of the most potential alternatives to FO, irrespective of the LC-PUFA biosynthesis capacity of fish.

In recent years, the application of AO as a FO substitute in aquafeed has attracted much attention. Previous studies on rainbow trout (*Oncorhynchus mykiss*) [41] and common carp (*Cyprinus carpio*) [42] have shown that the effect of AO was closely related to the substitution ratio, dietary DHA content, and fatty acid balance when AO was used alone or in combination with other oils. The growth results of this study showed that the FO replacement strategies with combination of AO and PO or RO did not significantly affect the growth performance of tiger puffer. Numerically, the weight gain ratio of the AO+PO group was even increased by 6% compared to the FO control group, indicating the potential of this oil combination in maintaining fish growth. Other studies have shown that the combined use of *Schizochytrium* sp. and soybean oil at a 4:3 ratio significantly improved the weight gain of Nile tilapia (*Oreochromis niloticus*) [43]. A mixture of 12% *Schizochytrium* sp. meal and flax oil maintained the normal health and growth while enhancing the PUFA content in fillets of sablefish (*Anoplopoma fimbria*) [44]. The dietary replacement of 80% FO solely with *Schizochytrium* sp. had no adverse effects on the growth and immunity of rainbow trout [45]. In shrimp diets, the complete replacement of FO with *Schizochytrium* meal and soybean oil did not adversely affect the growth performance or survival of white shrimp (*Litopenaeus vannamei*) [46]. However, the combined use of *Schizochytrium* biomass and olive oil in diets for seabream (*Sparus aurata*) altered the DHA:EPA ratio from 10:6 in the control group to 10:0.3, leading to significant reductions in growth and survival, likely due to the severe imbalance in the DHA:EPA proportions [47]. In contrast, complete FO replacement with *Schizochytrium* sp. oil in the diets of juvenile Atlantic salmon maintained the normal growth and survival, possibly because the DHA:EPA ratio remained balanced (2.6:0.2) despite the full replacement of FO with AO [14]. These findings underscore the critical importance of selecting appropriate lipid sources and optimizing their ratios to ensure balanced fatty acid profiles in diets, particularly in maintaining adequate DHA:EPA ratios, which directly influence the growth performance and physiological health of aquatic animals [48,49,50].

Besides growth performance, the muscle LC-PUFA content is also a major concern due to its close association with fillet quality. In this study, the combined use of AO and PO or RO increased the muscle DHA content but reduced the EPA and ARA contents. This closely reflected the dietary fatty acid contents [51]. Similar results were observed in studies on meagre (*Argyrosomus regius*), which were fed poultry/canola/algal oil blends [52], and those on Atlantic salmon fed DHA-rich *Schizochytrium sp.* meals [53] or microalgal oil extracted from *Schizochytrium* sp. [54]. Similar changing trends in response to dietary AO were also observed in bastard halibut (*Paralichthys olivaceus*) [55] and red seabream (*Pagrus major*) [56]. Most algae species from *Schizochytrium* [57], *Crypthecodinium* [58], and *Ulkenia* [59] are rich in DHA, but lack other LC-PUFAs, such as EPA and ARA. The average DHA content in *Schizochytrium limacinum* and *Thraustochytrium* is in the range of 30–40% of the total fatty acids [60]. Some species even contain as high as 53% DHA in the total fatty acids [61]. Suitable contents of EPA and ARA, as well as appropriate ratios between different LC-PUFAs in the diets, were also very important to fish growth, health, and fillet quality [62,63,64]. Therefore, if the residual EPA and ARA from dietary fishmeal were not enough for fish, the supplementation of other algae producing EPA and ARA, such as *Nannochloropsis gaditana* [65] and *Porphyridium purpureum* [66], would be necessary.

Although the liver in most fish species is not for human consumption, the fatty acid profile in the liver is important for fish physiology [67]. Similarly to muscle, the liver also showed higher DHA contents in the mixed-oil groups compared to the FO-C group, while the EPA content was lower than that in the FO-C group. However, compared to muscle, the contents of SFAs and MUFAs had significant differences among the groups in the liver, which better illustrated the differences in the fatty acid compositions of the experimental diets. This could be attributed to the fact that in tiger puffer, the liver serves as a primary organ for lipid storage, where dietary lipids are predominantly stored with minimal metabolic transformation [68]. Even in fish species that do not store lipids predominantly in the liver, such as largemouth bass [69], yellow perch (*Perca flavescens*) [70], and common carp *Cyprinus carpio* [71], the differences in the hepatic SFA and MUFA contents among the dietary groups still more closely reflected the inter-group differences in the dietary SFA and MUFA contents compared to muscle. Additionally, multiple studies have demonstrated that the SFAs and MUFAs in fish are primarily metabolized via β-oxidation to provide energy [72,73], while LC-PUFAs, such as DHA and EPA, are more likely to be retained for the formation of cell membranes and other physiological processes [74,75]. This hypothesis explains why the differences in SFAs and MUFAs in the diets were not reflected in the muscle, as these fatty acids were preferentially utilized for oxidative energy production, rather than long-term storage [76].

Regarding the body composition, the crude lipid content in the AO+PO group was significantly lower than that in the other two groups. Similar results have been observed in spotted knifejaw (*Oplegnathus punctatus*) [77], European sea bass (*Dicentrarchus labrax*) [78], and largemouth bass [79], where the addition of rapeseed oil in the diet was found to reduce the crude lipid content. Nevertheless, the dietary treatment in this study had little influence on other body composition parameters.

This study also assessed the effects of oil blends on the fish health status. From the serum indices, no significant differences were observed in any parameter analyzed except LDL-C, suggesting that the mixed oils had no significant adverse effects on the overall health status of the tiger puffer. The tissue histological analysis showed that the mixed-oil diets had no effect on the hepatic vacuolar area, but they significantly reduced the height and width of intestinal villi. Since the intestinal morphology and structure are important for nutrition intake and normal intestinal function [80], this result suggested that the combined use of AO and PO or RO may be harmful to fish bodies via impairing intestinal health. It was also observed that the addition of 0.3% and 0.6% AO in the experimental diets significantly reduced the villi height in Pacific white shrimp [81]. In other experiments, however, opposite results were observed. Feeding a diet supplemented with 3% AO increased the intestinal villi length in golden pompano, *Trachinotus ovatus* [82]. The experimental diets containing a mixture of soybean oil and AO increased the intestinal villi height and muscular thickness in Nile tilapia [83]. Another study on Nile tilapia showed that the addition of 1.2% AO to the diet did not affect the intestinal microvillar structure [69]. This discrepancy could be attributed to species-specific differences in intestinal structures and digestive physiology. High concentrations of n-3 LC-PUFAs (especially DHA) in the intestinal microenvironment may lead to the formation of lipid peroxidation end-products, such as 4-hydroxynonenal (4-HNE), which can directly disrupt the stability of tight junction proteins [84]. Additionally, as a natural ligand for PPARγ, excessive DHA intake may suppress the Wnt/β-catenin pathway, reducing enterocyte proliferation [85], thereby affecting villi morphology. Therefore, selecting appropriate AO inclusion levels in the diet could be crucial for maintaining the intestinal health of fish.

In the present experiment, the roles of oil blends in regulating the health status of tiger puffer were also further assessed at the level of molecular biology. The findings were in general agreement with the histopathological observations mentioned above. In the liver, the expression level of proinflammatory cytokine *ifn-γ* was significantly downregulated in the oil-blend groups, consistent with findings from previous studies [86,87]. The potential inflammation in the control group may be attributed to the use of plant-based protein sources in the basal diet, and the oil blends may be helpful to the alleviation of this potential inflammation. Furthermore, it was observed that the *tnf-α* expression was significantly downregulated in the AO+RO group. These effects may be mediated through the regulation of the nuclear factor E2-related factor 2/Kelch-like epichlorohydrin-associated protein-1 (Nrf2/Keap1) signaling pathway [88,89] which was evidenced in the present study. Also, the significant suppression of *acta2* gene expression suggested that the activation of hepatic stellate cells was suppressed [90], indicating that the AO mixtures may be effective in inhibiting the further development of fatty liver [91,92]. Together, the above evidence revealed the potential ability of AO blends in alleviating hepatic disorders. The intestinal barrier plays a crucial role in preventing the entry of pathogenic bacteria, toxins, and large molecules, thereby preserving intestinal homeostasis. *Claudin 18*, a vital component of tight junctions (TJs), regulates intercellular permeability and facilitates the transport of water, specific ions, and macromolecules. This protein also participates in cell adhesion, supporting the structural and functional integrity of epithelial and endothelial barriers [93,94,95]. In the intestine, significantly lower mRNA expression levels of *claudin 18* were detected in the AO+PO group. However, the simultaneous upregulation of *jam-a* and the downregulation of *ifn-γ* were observed in the AO+RO group. Combined with the observed changes in intestinal histology, these findings indicate that although the mixed-oil diet had a negative impact on the intestinal morphology, the impairment of intestinal function could be relatively minor. The ratio of algal oil to other oils in compound feeds can be systematically optimized, and the specific mechanism of action and safety threshold need to be further investigated by dose-effect experiments. In the muscle, the expression of *myod*, *myog*, *myf5*, and *myf6,* which are myogenic regulatory factors, was upregulated in the oil blend groups, indicating that the muscle cell differentiation and proliferation may be promoted [96,97,98]. Although the upregulation of both pro-apoptotic (*bax*) and anti-apoptotic (*bcl-2*) genes was observed, the upregulation of *bax* expression may represent an adaptive response of the organism. This mechanism could facilitate the clearance of damaged cells, support muscle tissue repair and regeneration, and thereby maintain the integrity of muscle function. Therefore, the DHA-rich AO blends may have the potential to promote muscle growth, which needs to be further verified.

## 5. Conclusions

In conclusion, the combination of *Schizochytrium* sp. oil with poultry oil or rapeseed oil had great potential as an alternative to fish oil in diets for tiger puffer (*Takifugu rubripes*), in terms of fish growth and fatty acid composition. However, since complete FO replacement with the oil mixtures may negatively affect the intestinal structure of tiger puffer, further investigation is required to establish the optimal inclusion levels and suitable ratios of *Schizochytrium* sp. oil to terrestrially sourced oils, in order to ensure the overall fish health status.

## Figures and Tables

**Table 1:** The formulation and proximate composition of the experimental diets (% dry matter basis).

Ingredients	FO-C	AO+PO	AO+RO
Fish meal	40.00	40.00	40.00
Poultry by-product meal	8.00	8.00	8.00
Soybean meal	11.00	11.00	11.00
Corn gluten meal	8.00	8.00	8.00
Wheat meal	17.68	17.68	17.68
Brewer’s yeast	5.00	5.00	5.00
Mineral premix ^a^	0.50	0.50	0.50
Vitamin premix ^a^	1.00	1.00	1.00
Monocalcium phosphate	1.00	1.00	1.00
L-ascorbyl-2-polyphosphate	0.20	0.20	0.20
Choline chloride	0.20	0.20	0.20
Betaine	0.30	0.30	0.30
Ethoxyquin	0.02	0.02	0.02
Calcium propionic acid	0.10	0.10	0.10
Soya lecithin	1.00	1.00	1.00
Fish oil	6.00		
*Schizochytrium* sp. oil ^b^		3.00	3.00
Poultry oil ^c^		3.00	
Rapeseed oil ^d^			3.00
Proximate composition			
Crude protein	49.98	49.98	49.98
Crude lipid	11.24	11.24	11.24
Ash	89.08	88.85	88.90
Energy kJ/g	20.96	20.91	20.89

^a^ Mineral premix and vitamin premix, which are designed specifically for marine fish, were purchased from Qingdao Master Biotech Co., Ltd. (Qingdao, China). ^b^ *Schizochytrium* sp. oil was purchased from Xiamen Huison Biotech Co., Ltd. (Xiamen, China). ^c^ Duck skin oil (poultry oil) was purchased from Shandong Haiding Agriculture & Animal Husbandry Co., Ltd. (Jinan, China). ^d^ Rapeseed oil was purchased from Shandong Luhua Group Co., Ltd. (Yantai, China).

**Table 2:** Fatty acid composition of fish oil, algal oil, poultry oil, rapeseed oil, and experimental diets (% total fatty acids).

Fatty Acid	Diet			Oil			
	FO-C	AO+PO	AO+RO	FO	AO	PO	RO
14:0	5.42	2.07	1.97	7.02	0.93	0.67	0.05
16:0	23.44	21.43	18.78	22.04	21.37	24.28	4.17
18:0	5.12	3.96	3.68	0.36	0.03	5.25	1.63
20:0	0.48	0.20	0.24	0.12	0.39	0.06	0.39
SFA	34.46	27.66	24.68	29.54	22.41	30.26	6.25
16:1n-7	0.62	0.16	0.17	0.45	0.04	0.13	0.06
18:1n-9	14.48	20.37	21.96	13.79	0.38	40.97	58.97
20:1n-9	1.47	0.48	0.53	0.06	ND	0.37	0.99
22:1n-9	0.28	0.22	0.20	0.70	0.10	ND	0.69
MUFA	16.86	21.23	22.85	15.0	1.75	41.47	61.05
18:2n-6	10.17	14.36	14.54	0.16	0.15	19.64	19.71
20:2n-6	0.21	0.14	0.13	0.03	0.27	0.15	0.06
20:4n-6	0.99	0.68	0.60	0.02	0.06	0.281	ND
n-6 PUFA	11.36	15.19	15.27	0.21	1.35	20.35	19.77
18:3n-3	1.43	1.62	2.21	0.50	0.13	0.71	0.12
20:5n-3	6.99	2.99	3.02	8.94	0.07	0.08	0.04
22:5n-3	1.05	0.55	0.53	0.98	0.22	0.05	ND
22:6n-3	11.47	19.86	20.02	13.83	59.44	0.20	0.06
n-3 PUFA	20.94	25.03	25.78	24.25	59.86	1.04	0.22
DHA/EPA	1.64	6.67	6.65	1.55	849.14	1.63	1.50

SFA: saturated fatty acid; MUFA: monounsaturated fatty acid; n-6 PUFA: n-6 polyunsaturated fatty acid; n-3 PUFA: n-3 polyunsaturated fatty acid; ND: nondetectable.

**Table 3:** Sequences of the primers used in this work.

Primer	Sequence (5′−3′)	GenBank Reference	PL (bp)
*myod*-F	TTCATCATCACACCGAGGCG	NM_001032769.1	126
*myod*-R	GTCGGTCCACGTTTGTAGTCT		
*myog*-F	ACGCTAATCAGTGGGTCTGC	XM_003973605.3	89
*myog*-R	TAACTCGTGGCTTCGACAGG		
*myf6*-F	GATCTGCAAGCGCAAATCGG	NM_001032771.1	116
*myf6*-R	CCACGGTCTTCCTCTTGAGC		
*myf5*-F	GGAGTCCTCTGTCCAACTGC	NM_001032770.1	84
*myf5*-R	CGCTGCTGTAAACTGCGTTC		
*bax*-F	ACCGTTCCCAGTGCAAATCT	XM_003964782.3	108
*bax*-R	TGGGAACACTTGAGCCCATC		
*bcl-2*-F	GGGCCGGATTATCGCTTTCT	XM_029830403.1	111
*bcl-2*-R	TATTCCGTCATCCACTCCGC		
*acta2*-6F	ATTCCTCGTCCCTGTGTGGTC	XM_029845356.1	125
*acta2*-6R	GGCATCATCTCCAGCGAAGC		
*il-1β*-F	CATCACCCGCTGACCATGAA	NM_001280090.1	103
*il-1β*-R	CATCCCTGAACTCGGTGCTC		
*il-8*-F	CCTGCGGAGCCTCGGAGTG	AB125645.1	145
*il-8*-R	TGACATCTTCAGAGTGGCAATGATCTC		
*tnf-α*-F	CTACTGGAACGGAAGGCAAGAGATG	AB183465.1	100
*tnf-α*-R	GATGCGGCTCAGCGTGTAGTG		
*ifn-γ*-F	CTGTGATGACTCTTGGGGCT	XM_029825554.1	147
*ifn-γ*-R	TGTACCGCTGACAGGAGTTG		
*claudin18*-F	GACACAAGGGTCTGTGGCAG	AY554344.1	112
*claudin18*-R	ATGATCATCAGGGCTCGCAC		
*jam-a*-F	CAAAAACGGCGTGCCTCTAC	XM_003971244.3	122
*jam-a*-R	CCGAGTCCGACCTTGATGTT		
*mlck*-F	GACACGACTGGCACGCAGATC	XM_029830403.1	170
*mlck*-R	CAGATGACTCCGATGCTCCACATG		
*rpl19*-F	GTCTCATCATCCGCAAACC	XM_003964816	132
*rpl19*-R	TCTCAGGCATACGAGCATT		
*rpl13*-F	GTAACAGGTCCACAGAATCCC	XM_003969972	117
*rpl13*-R	CCTCAGTGCTGTCTCCCTTC		
*keap1*-F	ACCGTGATGGAGGAATCGAGC	XM_029851225.1	123
*keap1*-R	TCAGCTTACCAGAACCGAGGG		
*nrf2*-F	ACGCATTCGACAAACACGAC	XM_003961827.3	106
*nrf2*-R	CCGTACACAGACTTCCCAGG		
*col1a2*-F	TGTTGGAGAGGGTGGAAAGC	XM_011609483.2	136
*col1a2*-R	GACTCCCATTGGACCCTGAG		

*myod*: myogenic differentiation antigen; *myog*: myogenin; *myf6*: myogenic factor 6; *myf5*: myogenic factor 5; *bcl-2*: b-cell lymphoma-2; *bax*: bcl-2-associated x; *acta2*: actin alpha 2; *il-1β*: interleukin—1β; *il-8*: interleukin 8; *tnf-α*: tumor necrosis factor alpha; *ifn-γ*: interferon gamma; *jam-a*: junctional adhesion molecule a; *mlck*: myosin light chain kinase; *rpl19*: ribosomal protein l19; *rpl13*: ribosomal protein l13; *keap1*: Kelch-like ECH-associated protein 1; *nrf2*: nuclear factor erythroid 2; *col1a2*: collagen type I alpha 2 chain; PL: product length.

**Table 4:** Effects of different lipid sources on the growth performance of juvenile tiger puffer (*Takifugu rubripes*).

Parameters	FO-C	AO+PO	AO+RO
Initial body weight (g)	23.85 ± 0.02	23.79 ± 0.01	23.81 ± 0.01
Final body weight (g)	67.87 ± 2.49	69.13 ± 2.95	66.20 ± 2.78
Weight gain (g)	44.02 ± 2.50	45.36 ± 2.95	42.40 ± 2.78
Weight gain ratio (%)	184.60 ± 10.53	190.79 ± 12.42	178.09 ± 11.69
Specific growth rate (%/d)	1.87 ± 0.07	1.90 ± 0.08	1.82 ± 0.07
Feed conversion ratio	1.30 ± 0.02	1.31 ± 0.05	1.37 ± 0.04
Survival (%)	97.33 ± 1.33	98.67 ± 1.33	94.67 ± 1.33
Hepatosomatic index (%)	9.66 ± 0.60	9.94 ± 0.62	9.09 ± 0.37
Viscerosomatic index (%)	14.22 ± 0.56	14.78 ± 0.58	13.58 ± 0.56
Condition factor (g/cm^3^)	3.15 ± 0.09	3.31 ± 0.08	2.97 ± 0.09

**Table 5:** Effects of different lipid sources on the body composition of juvenile tiger puffer (*Takifugu rubripes*).

Parameters	FO-C	AO+PO	AO+RO
**Whole fish**			
Crude protein (% w.w.)	18.84 ± 0.51	19.20 ± 0.54	19.62 ± 0.67
Crude lipid (% w.w.)	8.54 ± 1.07	9.41 ± 1.12	8.27 ± 0.65
Moisture (%)	70.32 ± 1.22	70.05 ± 1.18	69.40 ± 1.03
Ash (% w.w.)	3.11 ± 0.30	3.08 ± 0.29	3.27 ± 0.19
**Muscle**			
Crude protein (% w.w.)	18.96 ± 0.07	19.18 ± 0.23	18.86 ± 0.05
Crude lipid (% w.w.)	0.78 ± 0.01 ^b^	0.65 ± 0.01 ^a^	0.79 ± 0.03 ^b^
Moisture (%)	78.74 ± 0.27	78.80 ± 0.19	78.63 ± 0.26
**Liver**			
Crude protein (% w.w.)	3.84 ± 0.13	3.74 ± 0.43	4.11 ± 0.55
Crude lipid (% w.w.)	44.76 ± 2.75	46.35 ± 1.95	45.30 ± 2.00
Moisture (%)	30.93 ± 0.84	30.28 ± 0.34	30.10 ± 0.78

Data in the same row not sharing the same superscript letter are significantly different (*p* < 0.05). w.w.: wet weight.

**Table 6:** Effects of different lipid sources on the serum biochemical parameters of juvenile tiger puffer (*Takifugu rubripes*).

Parameters	FO-C	AO+PO	AO+RO
Triacylglycerol (mmol/L)	1.76 ± 0.19	1.53 ± 0.38	1.71 ± 0.33
Total cholesterol (mmol/L)	3.82 ± 0.61	3.32 ± 0.31	3.02 ± 0.36
High-density lipoprotein cholesterol (mmol/L)	4.97 ± 1.00	4.38 ± 0.47	4.23 ± 0.82
Low-density lipoprotein cholesterol (mmol/L)	0.93 ± 0.22 ^a^	1.13 ± 0.13 ^b^	0.88 ± 0.10 ^a^
Total bile acid (μmol/L)	1.96 ± 0.47	1.61 ± 0.39	1.62 ± 0.41
Malondialdehyde (nmol/mL)	10.17 ± 1.39	9.56 ± 1.51	9.29 ± 1.44

Data in the same row not sharing the same superscript letter are significantly different (*p* < 0.05).

**Table 7:** Effects of different lipid sources on the fatty acid compositions in the whole fish samples of juvenile tiger puffer (*Takifugu rubripes*) (% total fatty acids).

Fatty Acid	FO-C	AO+PO	AO+RO
14:0	2.89 ± 0.15 ^b^	1.32 ± 0.09 ^a^	1.49 ± 0.17 ^a^
16:0	22.65 ± 0.47 ^b^	20.81 ± 0.64 ^a^	19.82 ± 1.36 ^a^
18:0	6.65 ± 0.51 ^ab^	7.29 ± 0.71 ^b^	6.06 ± 0.80 ^a^
20:0	0.29 ± 0.02 ^b^	0.19 ± 0.03 ^a^	0.21 ± 0.03 ^a^
SFA	32.47 ± 0.82 ^b^	29.60 ± 0.58 ^a^	27.58 ± 2.13 ^a^
16:1n-7	0.30 ± 0.02 ^b^	0.20 ± 0.05 ^a^	0.16 ± 0.01 ^a^
18:1n-9	17.83 ± 1.27 ^a^	21.46 ± 1.81 ^b^	22.97 ± 1.39 ^b^
20:1n-9	1.56 ± 0.08 ^b^	1.05 ± 0.07 ^a^	1.23 ± 0.21 ^a^
22:1n-9	0.27 ± 0.03 ^b^	0.15 ± 0.02 ^a^	0.18 ± 0.07 ^a^
MUFA	19.95 ± 1.16 ^a^	22.85 ± 1.79 ^b^	24.54 ± 1.43 ^b^
18:2n-6	7.38 ± 0.20 ^a^	10.26 ± 0.60 ^b^	10.01 ± 0.59 ^b^
20:2n-6	0.44 ± 0.04	0.48 ± 0.03	0.53 ± 0.08
20:4n-6	0.71 ± 0.06	0.68 ± 0.17	0.54 ± 0.03
n-6 PUFA	8.52 ± 0.25 ^a^	11.41 ± 0.56 ^b^	11.07 ± 0.63 ^b^
18:3n-3	1.07 ± 0.06 ^a^	0.91 ± 0.07 ^a^	1.76 ± 0.44 ^b^
20:5n-3	4.46 ± 0.18 ^b^	2.02 ± 0.25 ^a^	1.98 ± 0.28 ^a^
22:5n-3	2.63 ± 0.10 ^b^	1.87 ± 0.08 ^a^	1.98 ± 0.11 ^a^
C22:6n-3	10.79 ± 0.46 ^a^	16.06 ± 0.87 ^b^	15.34 ± 0.71 ^b^
n-3 PUFA	18.94 ± 0.25 ^a^	20.83 ± 0.39 ^b^	21.11 ± 0.39 ^b^
DHA/EPA	2.42 ± 0.10 ^a^	8.06 ± 1.04 ^b^	7.87 ± 1.14 ^b^

Data in a row not sharing the same superscript letter are significantly different (*p* < 0.05). SFA: saturated fatty acid; MUFA: monounsaturated fatty acid; PUFA: polyunsaturated fatty acid.

**Table 8:** Effects of different lipid sources on the fatty acid compositions in the livers of juvenile tiger puffer (*Takifugu rubripes*) (% total fatty acids).

Fatty Acid	FO-C	AO+PO	AO+RO
14:0	3.06 ± 0.17 ^b^	1.46 ± 0.16 ^a^	1.38 ± 0.23 ^a^
16:0	22.08 ± 0.86 ^c^	20.86 ± 0.90 ^b^	17.71 ± 0.43 ^a^
18:0	6.45 ± 0.66 ^b^	6.11 ± 0.61 ^ab^	5.31 ± 0.70 ^a^
20:0	0.32 ± 0.02 ^c^	0.17 ± 0.01 ^a^	0.21 ± 0.01 ^b^
SFA	31.91 ± 1.20 ^c^	28.59 ± 0.96 ^b^	24.61 ± 0.41 ^a^
16:1n-7	0.29 ± 0.01 ^b^	0.15 ± 0.01 ^a^	0.14 ± 0.01 ^a^
18:1n-9	17.98 ± 1.21 ^a^	20.86 ± 0.84 ^b^	23.97 ± 1.17 ^c^
20:1n-9	1.64 ± 0.11 ^c^	1.01 ± 0.08 ^a^	1.26 ± 0.16 ^b^
22:1n-9	0.30 ± 0.04 ^c^	0.13 ± 0.02 ^a^	0.19 ± 0.04 ^b^
MUFA	20.21 ± 1.07 ^a^	22.15 ± 0.85 ^b^	25.57 ± 1.04 ^c^
18:2n-6	7.50 ± 0.28 ^a^	10.55 ± 0.18 ^b^	10.69 ± 0.38 ^b^
20:2n-6	0.44 ± 0.06	0.45 ± 0.04	0.50 ± 0.05
20:4n-6	0.57 ± 0.04	0.56 ± 0.13	0.44 ± 0.06
n-6 PUFA	8.51 ± 0.33 ^a^	11.56 ± 0.27 ^b^	11.63 ± 0.36 ^b^
18:3n-3	1.18 ± 0.04 ^b^	0.96 ± 0.07 ^a^	2.08 ± 0.06 ^c^
20:5n-3	4.32 ± 0.15 ^b^	2.22 ± 0.15 ^a^	2.63 ± 1.12 ^a^
22:5n-3	2.86 ± 0.08 ^b^	1.96 ± 0.04 ^a^	2.05 ± 0.07 ^a^
22:6n-3	10.72 ± 0.53 ^a^	17.23 ± 0.61 ^b^	16.62 ± 0.72 ^b^
n-3 PUFA	19.08 ± 0.28 ^a^	22.37 ± 0.30 ^b^	23.38 ± 0.32 ^b^
DHA/EPA	2.48 ± 0.18 ^a^	7.78 ± 0.36 ^b^	7.04 ± 2.04 ^b^

Data in a row not sharing the same superscript letter are significantly different (*p* < 0.05). SFA: saturated fatty acid; MUFA: monounsaturated fatty acid; PUFA: polyunsaturated fatty acid.

**Table 9:** Effects of different lipid sources on the fatty acid compositions in the muscles of juvenile tiger puffer (*Takifugu rubripes*) (% total fatty acids).

Fatty Acid	FO-C	AO+PO	AO+RO
14:0	0.59 ± 0.06 ^b^	0.34 ± 0.05 ^a^	0.36 ± 0.00 ^a^
16:0	24.23 ± 0.58	25.36 ± 1.73	23.01 ± 0.92
18:0	7.88 ± 0.29 ^ab^	8.40 ± 0.28 ^b^	7.32 ± 0.21 ^a^
20:0	0.22 ± 0.02 ^b^	0.15 ± 0.04 ^a^	0.18 ± 0.01 ^ab^
SFA	32.92 ± 0.88	34.19 ± 1.97	30.86 ± 1.00
16:1n-7	1.03 ± 0.07 ^b^	0.76 ± 0.06 ^a^	0.73 ± 0.08 ^a^
18:1n-9	13.41 ± 0.86	13.56 ± 1.33	14.59 ± 0.68
20:1n-9	0.77 ± 0.05	0.67 ± 0.46	0.54 ± 0.03
MUFA	15.44 ± 0.80	15.00 ± 1.48	15.86 ± 0.67
18:2n-6	7.72 ± 0.02	8.64 ± 0.36	8.59 ± 0.66
20:2n-6	0.39 ± 0.05	0.45 ± 0.06	0.47 ± 0.04
20:4n-6	2.50 ± 0.05 ^b^	1.75 ± 0.21 ^a^	1.65 ± 0.02 ^a^
n-6 PUFA	10.61 ± 0.12	10.84 ± 0.11	10.71 ± 0.69
18:3n-3	0.41 ± 0.02 ^b^	0.20 ± 0.03 ^a^	0.55 ± 0.13 ^b^
20:5n-3	5.91 ± 0.44 ^b^	2.36 ± 0.75 ^a^	2.07 ± 0.41 ^a^
22:5n-3	2.34 ± 0.05 ^b^	1.12 ± 0.06 ^a^	1.17 ± 0.02 ^a^
22:6n-3	21.91 ± 0.71 ^a^	23.42 ± 0.45 ^a^	26.18 ± 0.30 ^b^
n-3 PUFA	30.57 ± 0.89	27.12 ± 0.92	29.97 ± 0.57
DHA/EPA	3.71 ± 0.12 ^a^	10.53 ± 2.63 ^b^	13.01 ± 2.70 ^b^

Data in a row not sharing the same superscript letter are significantly different (*p* < 0.05). SFA: saturated fatty acid; MUFA: monounsaturated fatty acid; PUFA: polyunsaturated fatty acid.

## Data Availability

Raw data supporting the conclusions of this manuscript will be made available by the authors, without undue reservation, to any qualified researcher.

## Abbreviations

Non-gene name	
LC-PUFA	long-chain polyunsaturated fatty acids
EPA	eicosapentaenoic acid
DHA	docosahexaenoic acid
ARA	arachidonic acid
FO	fish oil
AO	algal oil
SFA	saturated fatty acid
MUFA	monounsaturated fatty acid
PO	poultry oil
RO	rapeseed oil
RT-qPCR	real-time quantitative polymerase chain reaction
Gene name	
*myod*	myogenic differentiation antigen
*myog*	myogenin
*myf6*	myogenic factor 6
*myf5*	myogenic factor 5
*bax*	*bcl-2*-associated x
*bcl-2*	b-cell lymphoma-2
*acta2*	actin alpha 2
*il-1β*	interleukin—1β
*il-8*	interleukin 8
*tnf-α*	tumor necrosis factor alpha
*ifn-γ*	interferon gamma
*jam-a*	junctional adhesion molecule a
*mlck*	myosin light chain kinase
*rpl19*	ribosomal protein l19
*rpl13*	ribosomal protein l13
*keap1*	Kelch-like ECH-associated protein 1
*nrf2*	nuclear factor erythroid 2
*col1a2*	collagen type I alpha 2 chain
